# Interplay between success and patterns of human collaboration: case study of a Thai Research Institute

**DOI:** 10.1038/s41598-020-79447-z

**Published:** 2021-01-11

**Authors:** Antonio Maria Fiscarelli, Matthias R. Brust, Roland Bouffanais, Apivadee Piyatumrong, Grégoire Danoy, Pascal Bouvry

**Affiliations:** 1 Luxembourg Centre for Contemporary and Digital History (C2DH), University of Luxembourg, Esch-sur-Alzette, Luxembourg; 2 Interdisciplinary Centre for Security, Reliability and Trust (SnT), University of Luxembourg, Esch-sur-Alzette, Luxembourg; 3 Department of Mechanical Engineering, University of Ottawa, Ottawa, Canada; 4 Department of Computer Science (FSTM/DCS), University of Luxembourg, Esch-sur-Alzette, Luxembourg; 5 NSTDA Supercomputer Center (ThaiSC), National Electronics and Computer Technology Center (NECTEC), Pathum Thani, Thailand

**Keywords:** Computer science, Computational science

## Abstract

Networks of collaboration are notoriously complex and the mechanisms underlying their evolution, although of high interest, are still not fully understood. In particular, collaboration networks can be used to model the interactions between scientists and analyze the circumstances that lead to successful research. This task is not trivial and conventional metrics, based on number of publications and number of citations of individual authors, may not be sufficient to provide a deep insight into the factors driving scientific success. However, network analysis techniques based on centrality measures and measures of the structural properties of the network are promising to that effect. In recent years, it has become evident that most successful research works are achieved by teams rather than individual researchers. Therefore, researchers have developed a keen interest in the dynamics of social groups. In this study, we use real world data from a Thai computer technology research center, where researchers collaborate on different projects and team up to produce a range of artifacts. For each artifact, a score that measures quality of research is available and shared between the researchers that contributed to its creation, according to their percentage of contribution. We identify several measures to quantify productivity and quality of work, as well as centrality measures and structural measures. We find that, at individual level, centrality metrics are linked to high productivity and quality of work, suggesting that researchers who cover strategic positions in the network of collaboration are more successful. At the team level, we show that the evolution in time of structural measures are also linked to high productivity and quality of work. This result suggests that variables such as team size, turnover rate, team compactness and team openness are critical factors that must be taken into account for the success of a team. The key findings of this study indicate that the success of a research institute needs to be assessed in the context of not just researcher or team level, but also on how the researchers engage in collaboration as well as on how teams evolve.

## Introduction

Networks are used to model different systems such as biological ones (Jeong et al.^1^), the world wide web (Réka, Hawoong and Barabási^2^), organizations and societies. Social Network Analysis is a truly interdisciplinary domain that has gained traction due to the recent access to large-scale datasets (“Big Data”) available online. A social network can be described as a collection of actors that are connected to each other if they form some sort of relationship^3^. A collaboration network is a particular social network where nodes represent individuals belonging to an institution/organization/company and edges represent collaboration and/or interaction between individuals^4^. Networks of collaboration are notoriously complex and the mechanisms underlying their evolution, although of high interest, are still not fully understood. In particular, collaboration networks can be used to model the interactions between scientists and analyze the circumstances that lead to successful research.

Scientific success and productivity have a skewed distribution^5^. Elmacioglu and Lee^6^ have shown how a small fraction of authors publish a large number of papers. Seglen, similarly, has shown that a small portion of articles collect most of the citations^5^. Newman and Girvan^7^ uncovered that the distance between scientists in a collaboration network is typically small (i.e. small world property) and that, for most scientists, all paths between them and other scientists go through only one or two of their collaborators (the so-called funneling effect). In the work of Backstrom et al.^8^, attention was drawn to the evolution of communities in collaboration networks and the structural features that influence the decision of individuals to join a community. Longitudinal analyses were also performed by Huang et al.^9^ and Bird et al.^9^ to find the structural differences between topical areas, identified as communities.

The factors that define a successful collaboration are various and of different nature. Mattessich and Monsey, in their book^10^, review the existing literature on which factors influence the success of collaborations, as well as what measures can be taken to enhance fruitful collaboration. They define collaboration as “a mutually beneficial and well defined relationship shared into by two or more individuals or organisations to achieve common goals”. They classify these factors in the following categories: environment, membership, structure, communication, process and resources. Amongst these many factors, the ones related to communication are worth mentioning. They found that established informal and formal communication links play an important role in successful collaborations: members that establish inter-personal relationships produce better results when working on a common project. Also, members that engage in open and frequent communication favour the transmission of information within and outside the group.

One of the biggest challenges is the one to find any links between patterns of collaboration and scientific success. Authors, with the increasing pressure to publish more, tend to seek for more collaborations^6^. Collaborators can have a large effect on a researcher’s career and choosing the right collaborators can have long-term implications on access to knowledge and resources. Borjas and Doran^11^ argue that spillover exists in three dimensions: idea (working on the same topic), geographic (working in the same department/university/region) and collaboration (co-authorship). They put the spillover hypothesis to the test, which states that emigration of researchers in any of these dimensions would lead to a reduced productivity for the researchers left. They found that spillover does not affect the average researcher, but it will affect researchers who lose a regular coauthor. Petersen^12^ shows that researcher’s collaboration patterns consist of high turnover collaborations, identified in weak ties, as well as steady and frequent collaborations with “life partners”, identified in super ties. He presents the “apostle effect”, that illustrates the advantage of strong and committed relationships. A longitudinal analysis was reported by Abramo et al.^13^ to find a causal effect between collaboration patterns and performance. They found that researchers who moved higher in rank tend to have fewer intramural collaborations, while favoring international ones, thereby leading to publications of higher impact. Feng and Kirkley^14^ analyzed the link between researchers’ neighborhood structure and academic performance. They found that researchers who collaborate with many teams and work on several projects have a longer career and are highly performing. Cross et al.^15^ found that even informal networks, such as friendship, contribute positively to job satisfaction and performance. They argue that, even if these sort of networks cannot be directly controlled by management, they can still be affected by factors such as hierarchical levels, office location, project staffing and so on.

Petersen et al.^16^ underline the urgency of defining new performance measures for individuals and groups. Conventional metrics, based on number of publications and number of citations, may not be sufficient to provide a deep insight into the factors driving scientific success. Seglen^5^, for example, argues that the skewed distribution of authors’ citations and the different citation practices in each field of research make citation count not suitable to evaluate researchers’ success. Instead, network analysis techniques based on centrality measures could be used to shed a new light on some mechanisms of success. Centrality metrics were first used by Bavelas^17,18^ who linked these metrics to team performance and productivity. Uddin et al.^19^ used both correlations and regression methods to link centrality measures and performance, showing that scientists that cover central positions in the network (high betweenness centrality) and have many collaborators (high degree centrality) are also highly cited. Similarly, Sarigöl et al.^20^ showed that centrality measures (degree centrality, eigenvector centrality, betweenness centrality and k-core centrality) of authors at the time of publication are good predictors of their citation count in the following 5 years.

In recent years, interest moved from individuals to teams. It has become more and more apparent that the most successful research is carried out by teams rather than single researchers^21^. It has been found that, over the past 50 years, teams increasingly dominate solo authors in the knowledge production. In addition, teams are more likely to produce high impact research in academia as well as in the private sector^22^. Uzzi et al.^23^ also found that teams are 37.7% more likely than solo authors to bring novelty into established knowledge domains, and that papers of this type are twice as likely to be highly cited. Petersen et al.^16^ show that scientific productivity is related to researchers’ visibility and team efficiency. In fact, team works have higher impact due the larger number of coauthors that will introduce their work to more peers.

For all these reasons, researchers have become interested in studying the dynamics of social groups, which consists of series of changing events such as formation and dissolution of teams. Guimera et al.^24^ proposed a method for group evolution discovery, based on a similarity measure between groups, and showed that the most successful teams are the ones that have a large core of established members who actively seek new collaborations. Palla et al.^25^ proposed another technique, based on the Clique Percolation Method, to investigate the evolution of groups over time. It was found that large and small groups behave differently. Specifically, large groups have a higher lifespan when turnover is high, meaning that there is a constant flow of newcomers, while small groups are better off when their composition remains unchanged over time. On the other hand, Kenna and Berch^26^ propose the notion of critical mass, for which research quality increase with group size only up to a maximum size referred to as critical mass. Goa et al.^27^ proposed a different method, based on central nodes identification, to study patent classes of similar technologies. Finally, Reagans and Zuckerman^28^ put to the test two hypotheses: the closure view of social capital and the structural hole view on social capital. The closure view of social capital states that teams that experience more frequent communication among their members (higher density) can achieve higher productivity^29^. The structural holes view on social capital, instead, states that teams that experience more frequent communication among members with different attributes (more heterogeneous) achieve a higher level of productivity^30^. They found both hypotheses to hold true and, particularly, that team density is more advantageous for heterogeneous teams.

In this study, we use a dataset from the National Electronics and Computer Technology Center (NECTEC) in Thailand,where researchers collaborate on different projects and team up to produce a range of artifacts (intellectual properties, prototypes and scientific articles). For each artifact, a score that measures quality of research is available and shared between the researchers that contributed to its creation, according to their percentage of contribution. We build a collaboration network where researchers are connected if they worked together on one or more artifacts. We find that, at individual level, centrality metrics are linked to high productivity and quality of work, suggesting that researchers who cover strategic positions in the network of collaboration are more successful. At the team level, we show that the evolution in time of structural measures are also linked to high productivity and quality of work. This result suggests that variables such as team size, turnover rate, team compactness and team openness must be taken into account for the success of a team.

What makes this study original is the analysis based on a score that measures research quality rather than quantity (e.g. number of output and citation count), as well as a new set of structural metrics that help identify features of teams that are linked to success. Even though the analysis is limited to a rather small dataset, we believe the dataset is rich in information, especially for the availability of a quality score, which is often neglected or is just not available for analysis in other studies. Nonetheless, the methodology adopted can be extended to larger datasets/networks. The key findings of this study indicate that the success of a research institute needs to be assessed in the context of not just researcher or team level, but also on how the researchers engage in collaboration as well as on how teams evolve.

## Results

**Figure 1:**
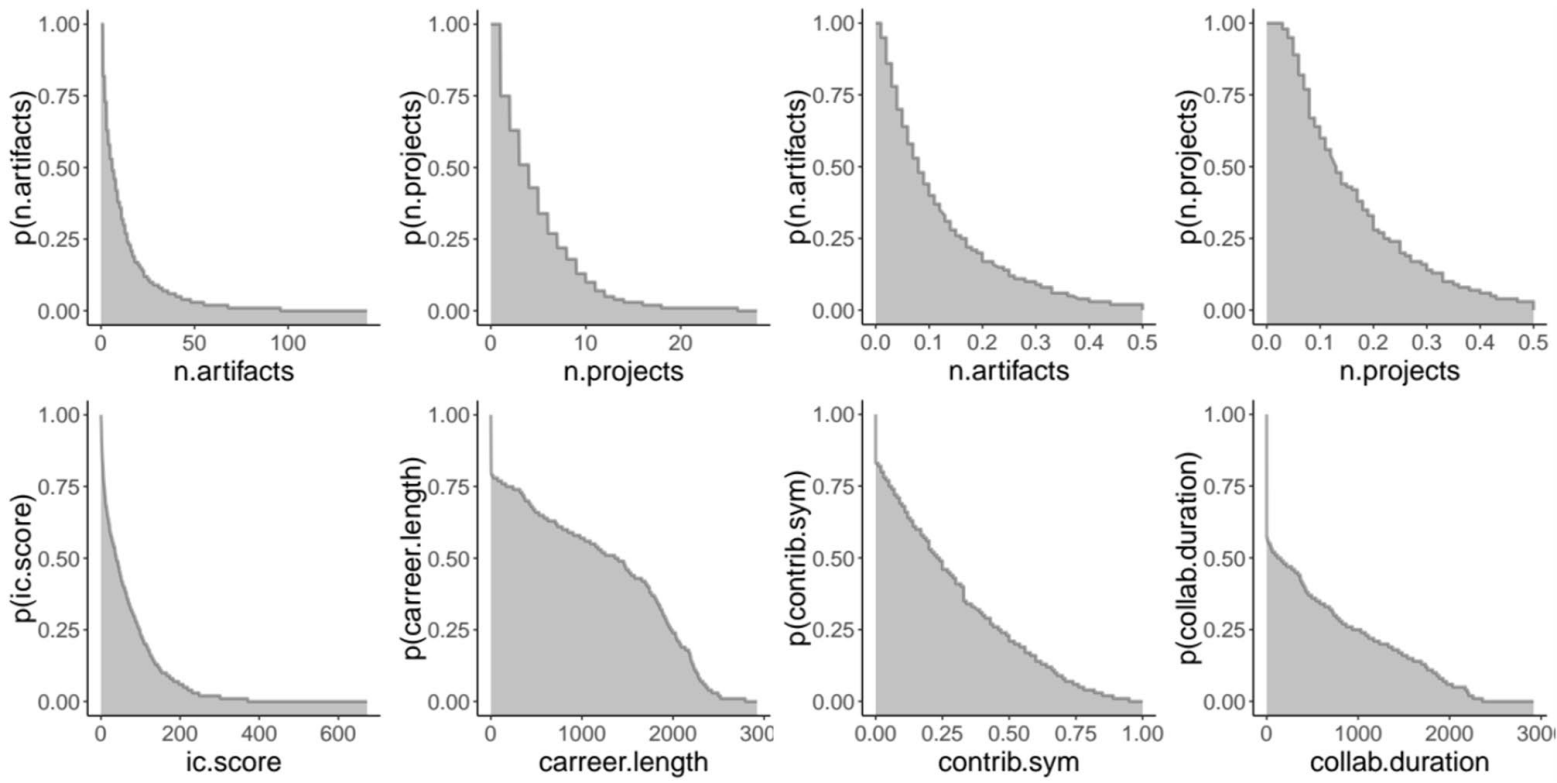
Node (left) and edge (right) attribute distributions for the NECTEC researchers network. The *x*-axis shows the range of an attribute value on a linear scale (“Methods” section for definition of these attributes). The *y*-axis shows the probability that a node has that attribute value or greater. The attribute career.length and collab.duration are measured in days.

First, we analyzed the distribution for some of the attributes for the NECTEC researchers network. For node attributes we considered number of artifacts, number of projects, Intellectual Capital (IC) Score and career length, while for edge attributes we considered number of artifacts, number of projects, contribution symmetry and length of collaboration. Results are shown in Fig. 1. As can be noticed, few researchers produce a large number of artifacts, while most researchers produce only a few. Number of projects and IC score are distributed in a similar manner, where few researchers are able to carry out many projects at the same time, while having a high IC score. Similar results can be observed for edge attributes. These results reflect the skewed distribution of scientific productivity. Regarding the career lengths, most researchers are active for no more than 75% of the entire time frame, with very few exceptions.

**Figure 2:**
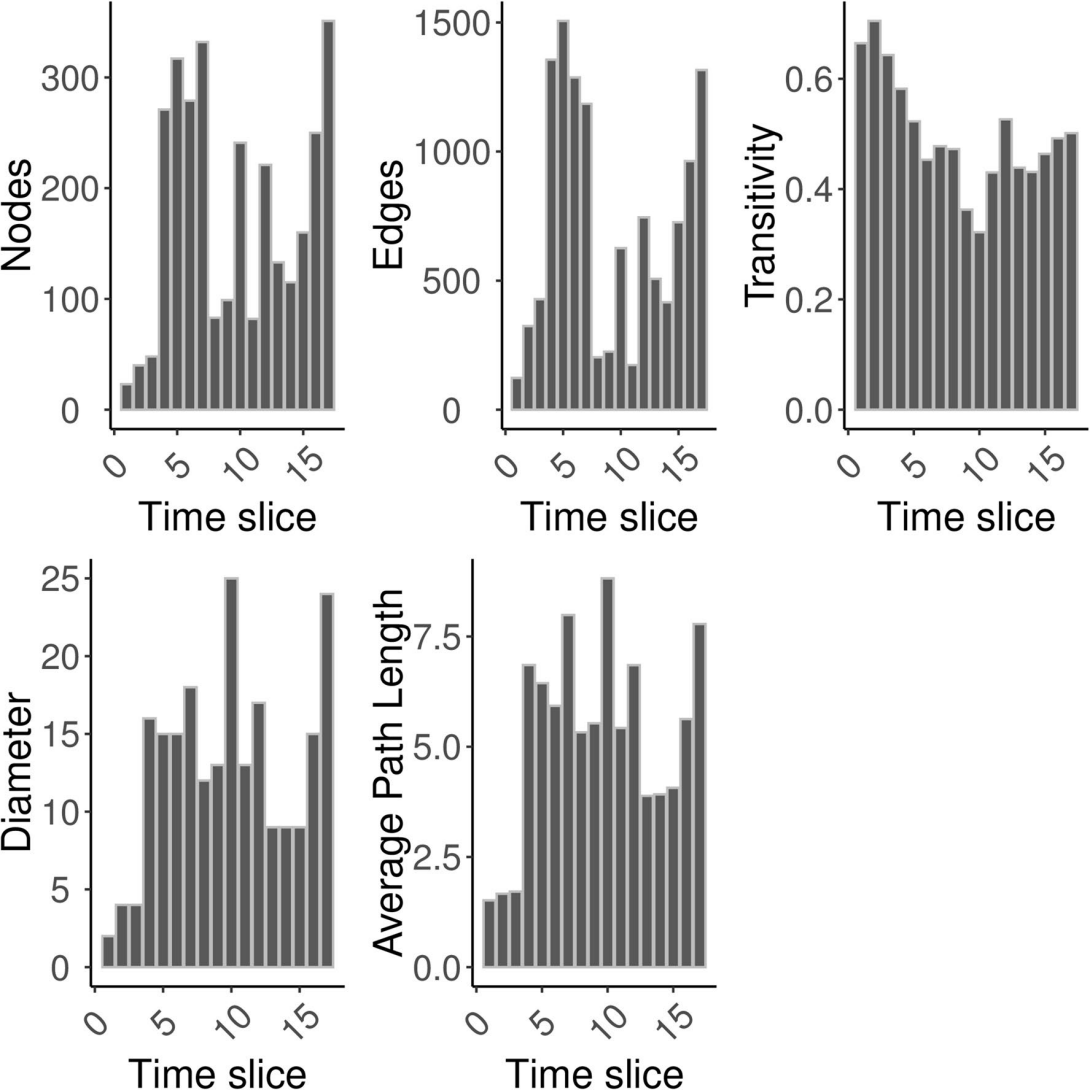
Global metrics for the NECTEC researchers network.

We then analysed the evolution of the researchers network over time by computing some global metrics such as number of nodes, number of edges, global transitivity, diameter and average path length for each time slice. Figure 2 shows the results. In general, the first time slices contain very few nodes. This is because of a few projects starting off early. After that, more researchers become active for few time slices, but time slices 8–12 show again lower activity. Higher diameter and average path length seem to be indicators of a less active and less dense network, while transitivity reaches its lowest value.

### Analysis of researchers’ collaboration patterns and performance

**Figure 3:**
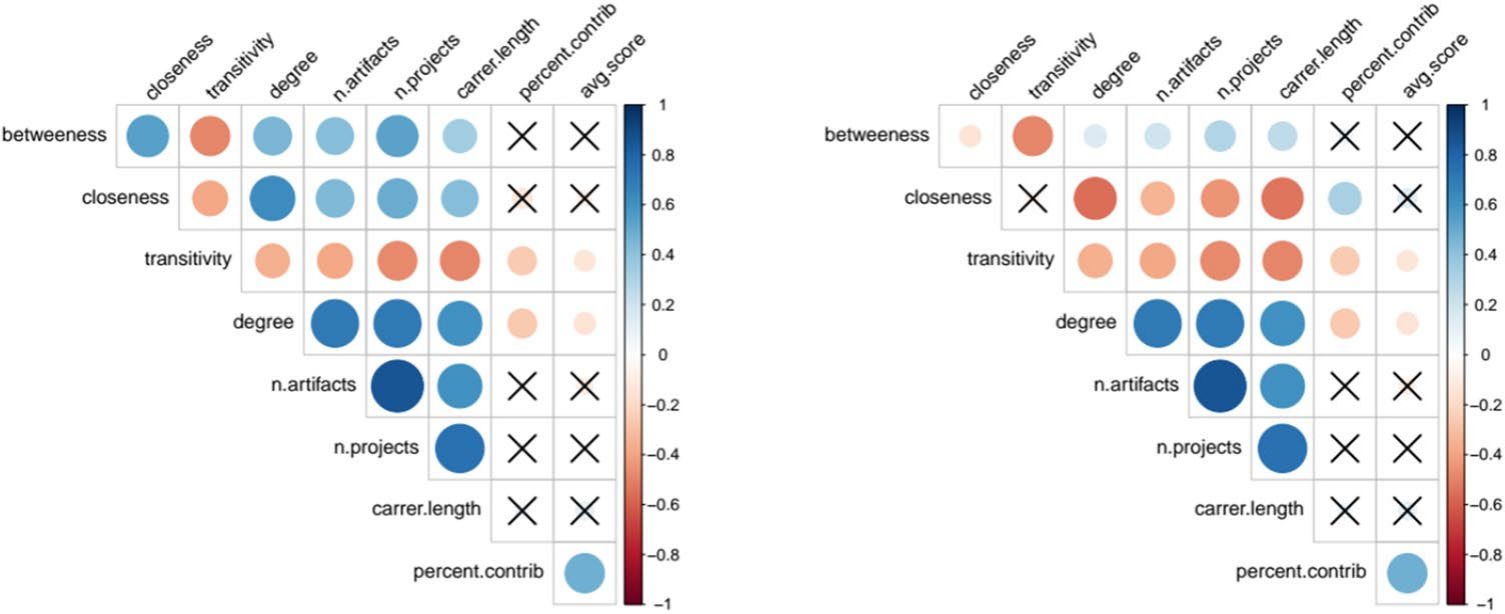
Correlation matrix for the NECTEC researchers network. Blue indicates positive correlation and red indicates negative correlation. Color intensity indicates the strength of correlation. Insignificant coefficients, according to *p* value $p = 0.001$, are marked with a cross. On the right, betweenness and closeness centrality have been normalized by degree centrality.

We computed correlations between a combination of performance metrics and local network metrics. We used number of artifacts, number of projects and career length as productivity metrics, as well as average IC score as quality metric (see “Methods” section). For local network metrics, we use betweenness, closeness, degree and transitivity. Figure 3 (left) shows the correlation matrix for the NECTEC researchers network. Number of artifacts, number of projects and career length correlate positively with degree, since researchers that are more productive or have a longer career also have more opportunity to engage in new works and collaborations. Number of artifacts, number of projects and career length also correlate positively with betweenness and closeness centrality. Therefore researchers that cover more central positions in the network appear to be more productive. Finally, number of artifacts, number of projects and career length correlate negatively with transitivity. The average IC score shows a different trend. It is not correlated with betweenness and closeness centrality, and it correlates negatively with degree and transitivity. This result suggests that researchers that have fewer collaborations at the same time produce higher quality work. As it can be noticed, centrality metrics show similar correlations with other metrics. Therefore, we decided to disentangle the link between them by normalising betweenness and closeness by degree centrality. There, Fig. 3 (right) shows the same correlation analysis, where betweenness and closeness centrality are normalized. Results are slightly different. Betweenness centrality is still positively correlated with number of artifacts, number of projects and career length, even when normalized by degree. Closeness centrality, instead, is negatively correlated to the same quantities. This shows that high degree centrality alone cannot explain high productivity metrics such as number of artifacts/projects and long career length, and betweenness still plays an important role. Not only is the number of connections relevant, but also the position of a researcher in the network.

### Orbit analysis

**Figure 4:**
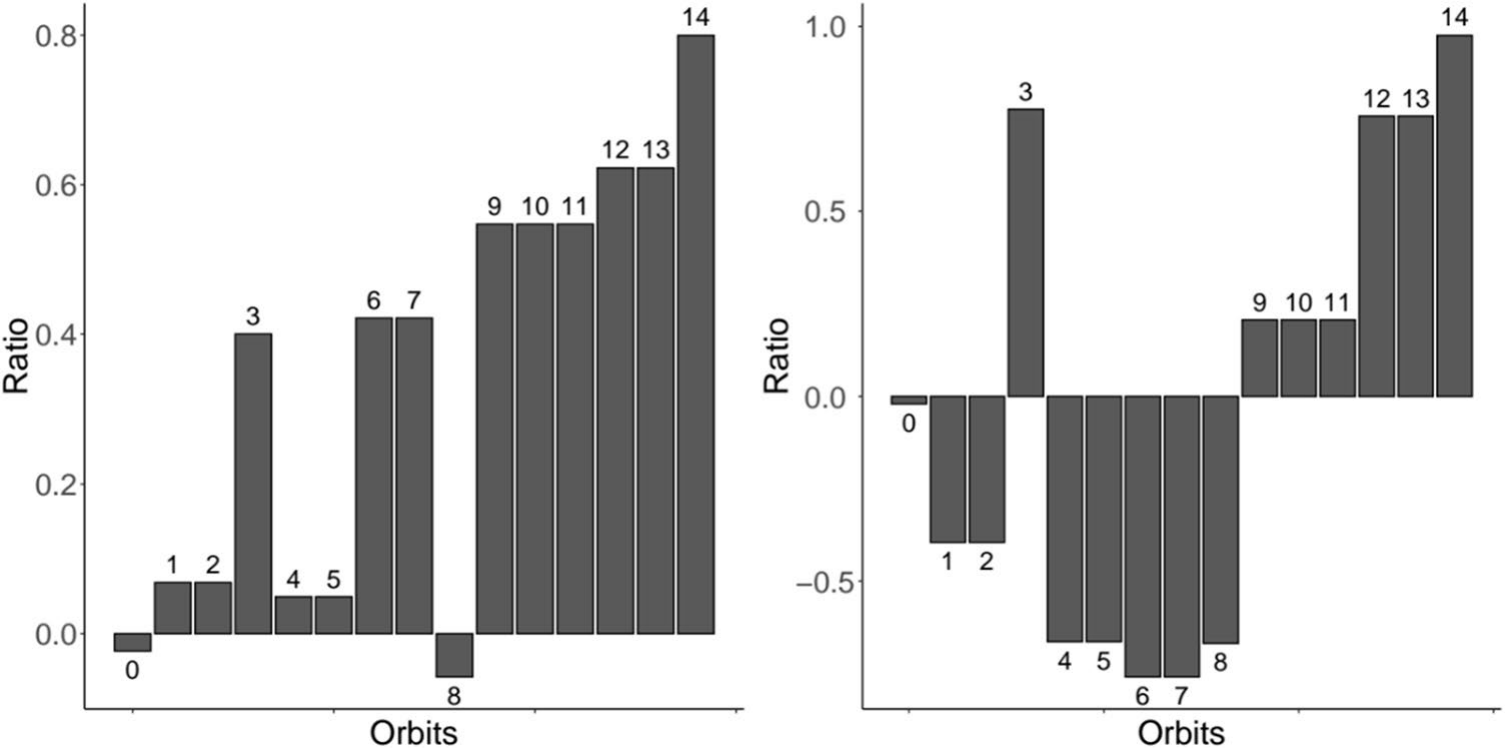
Orbit analysis, up to 4 nodes, for the researchers network. Bars go upwards if an orbit is more likely to be found in the real network compared to the null model. Left uses the Watts–Strogatz model as null model, while right uses Barabási model.

We used orbit analysis to analyse the structure of researchers’ neighborhood. Figure 4 shows the average orbit count for the NECTEC researchers network for graphlets up to size four, when compared to the Watts–Strogatz and Barabási models. In the first case, it can be noticed that orbits that are part of triangle-like graphlets (3, 10, 11, 12, 13, 14) and star-like graphlets (6, 7, 9, 10, 11) are more likely to be found in the researchers network, compared to a small world model. On the other hand, orbits that are part of chain-like graphlets (0, 1, 2, 4, 5, 8) are neither over or under represented. In the second case, orbits that are part of triangle-like graphlets are more likely to be found in the researchers network when compared to a preferential attachment model, while star-like graphlets and chain-like graphlets are under represented.

In a difference perspective, triangle-like graphlets are over represented in the researchers network, when compared to both null model. This is a natural consequence of the high level of transitivity in the network, and it shows that transitivity for this network is not merely a consequence of small world phenomenon or preferential attachment. This is also a consequence to the fact that researchers are clustered within the same projects. Star-like graphlets, instead, are highly represented in the network only when compared to the Watts–Strogatz models. This means that preferential attachment is sufficient to explain the presence of these types of graphlets. This may be due to the presence of high position researcher or project directors, that cover central positions in the network and collaborate with many other researchers on disjoint projects, that are therefore not connected to each other. Finally, star-like graphlets are under represented only when compared to the Barabási model. Since star-like configurations are a direct consequence of preferential attachment, their low presence in the researcher network shows that preferential attachment is not very strong. To summarize, triangle-like graphlets are more represented than star-like graphlets in the researcher network. This means that transitivity, more than preferential attachment, is the main force that drives researchers to connect. In other words, researchers that are choosing their collaborators prefer a common peer to a highly skilled person. Of particular interest, the graphlet $G_6$ (brokerage) is also highly represented in the network when compared to both models. Within this graphlet, orbit 11 serves as bridge between two connected nodes (orbit 10) and a single one (orbit 9). This orbit is specifically found in collaboration networks where actors that cover such position serve as “mediators” between different teams/groups.

**Figure 5:**
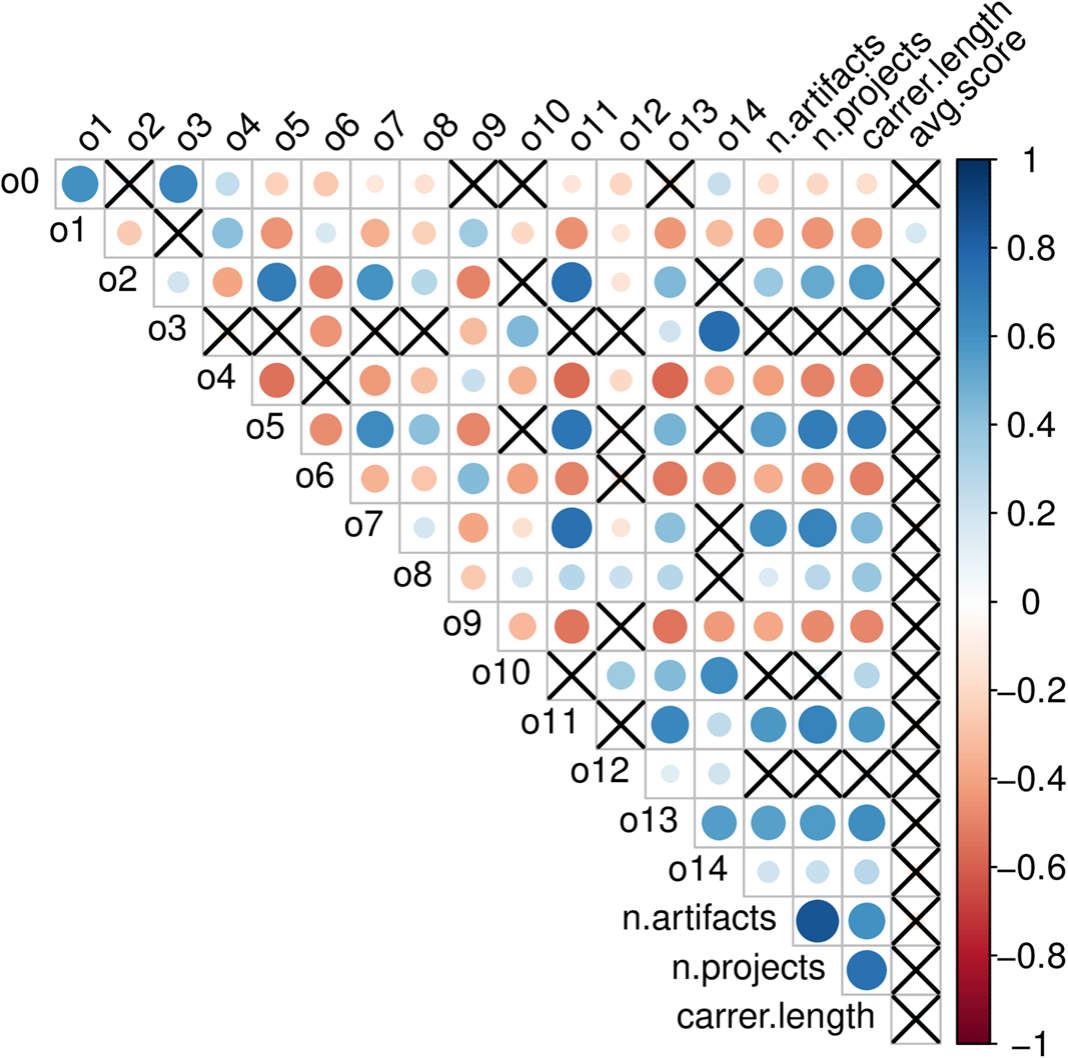
Correlation matrix for the NECTEC researchers network. Blue indicates positive correlation and red indicates negative correlation. Color intensity indicates the strength of correlation. Insignificant coefficients, according to *p* value $p = 0.001$, are marked with a cross.

We then proceeded by computing correlations between performance metrics and orbit counts. We used number of artifacts, number of projects and career length as productivity metrics and average IC score as quality metric (see “Methods” section). Figure 5 shows the correlation matrix for the NECTEC researchers network. The orbits that are positively correlated with productivity metrics are orbit 2, 5, 7, 8, 11, 13, 14. There is no specific graphlet type that stands out (triangle, star or chain). At the same time, degree is also not the key factor. In fact, among all orbits with degree two, some correlate positively (orbits 2, 5, 8, 12) and some others do not (orbits 3, 10). It can be noticed, instead, that all orbits that correlate positively with productivity metrics are the ones that, within the graphlet, are the most central. This result is in agreement with the previous correlation analysis, which showed that central nodes (nodes with high betweenness centrality) are more productive. For what concerns the IC score, instead, there is no correlation with any of the orbits analyzed.

### Team evolution

**Figure 6:**
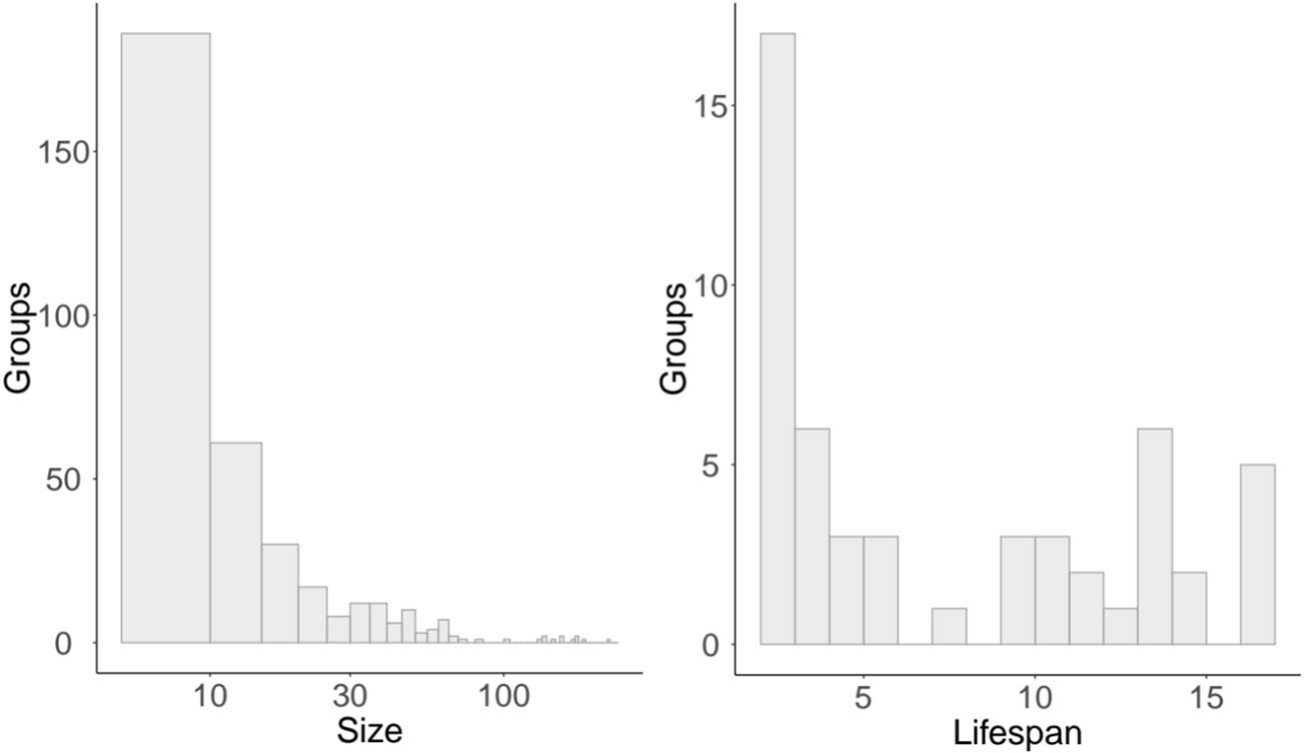
Team size and lifespan (measured in time slices) distribution for the temporal network. The diagram for team size has a logarithmic *x*-axes.

First, we analyzed the distribution of team size and lifespan over time for the NECTEC researchers network. Figure 6 shows the results. The network is composed of a few large teams, while the majority of the teams are small (i.e. less than ten members). Team lifespan, instead, shows a bimodal distribution, with most teams having either a short or long lifespan.

**Figure 7:**
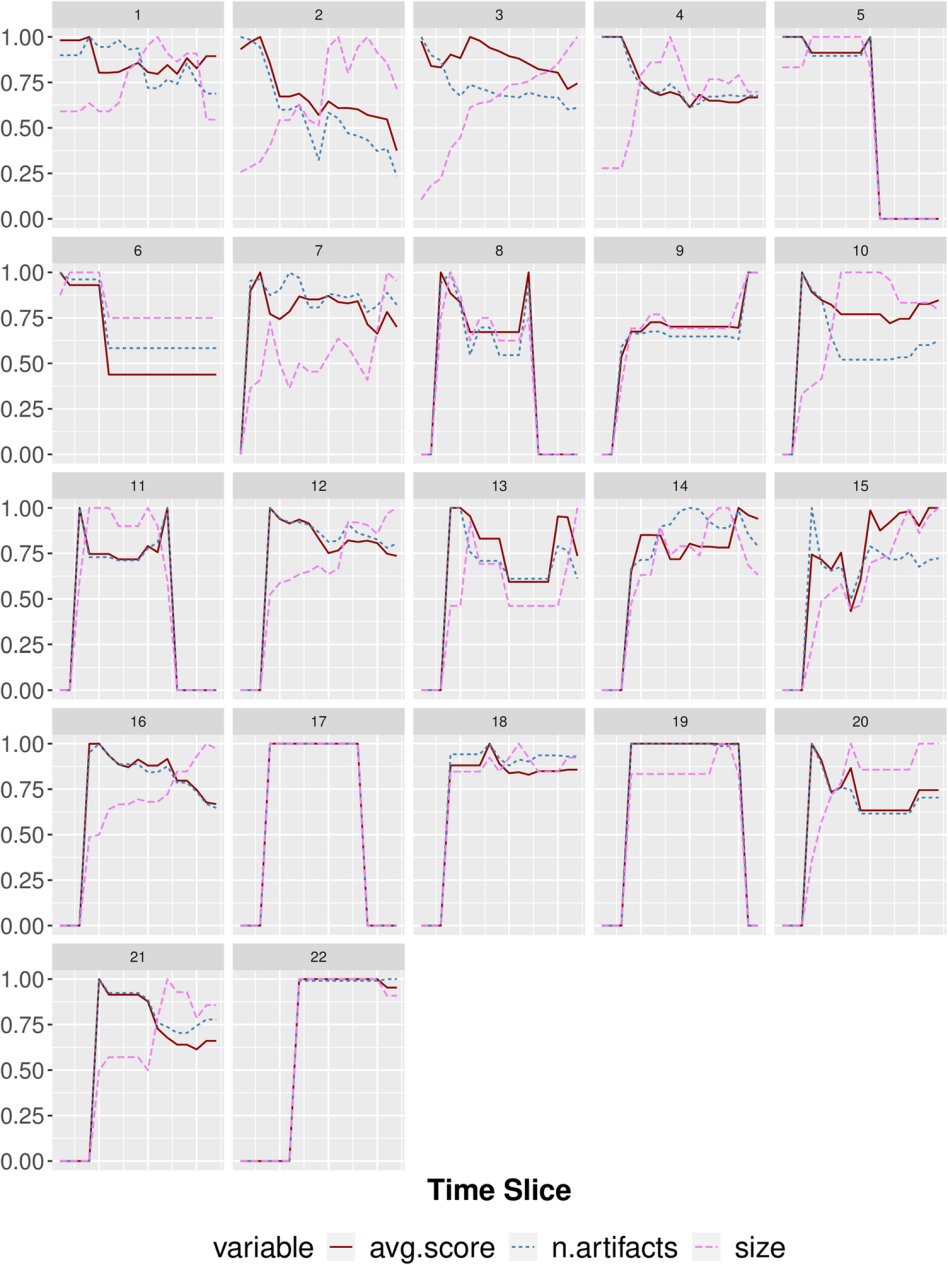
Each plot shows team size, number of artifacts and average IC score of a team over time. Only teams with lifespan ten or larger are considered. Time slice on the *x*-axes and observed variables (in percentage w.r.t maximum value) on the *y*-axes. Color and line type represents size, number of artifacts and average IC score.

We looked at the evolution of teams over time. We tracked size (team structure metrics), as well as number of artifact and average IC score produced by all researchers (team performance metrics). For this analysis, we only considered teams whose lifespan was ten or higher, hence focusing on teams that fall within the second mode of the lifespan distribution. Figure 7 shows all the results. Generally, all teams start off as small and grow in size in the successive time slices, they then either keep their size or shrink towards the end of their life time. As it can be noticed, there are very different teams. For some teams (5, 6, 7, 8, 9, 11, 12, 13, 14, 15, 17, 18, 19, 22), an increase/decrease in size is followed by an increase/decrease in number of artifacts and IC score. This can be the case when experienced researchers join or leave a team, since their presence/absence highly affects the overall score of a team. In other cases (1, 2, 3, 4, 10, 16, 20, 21), an increase in size is followed by a decrease in number of artifacts and IC score. This can be the case when newcomers join an already established team, for which they do contribute in size but not to the overall score. Finally, an IC score that is significantly higher than the number of papers (2, 3, 10, 15) is an indicator of teams that produce, on average, high quality work.

**Figure 8:**
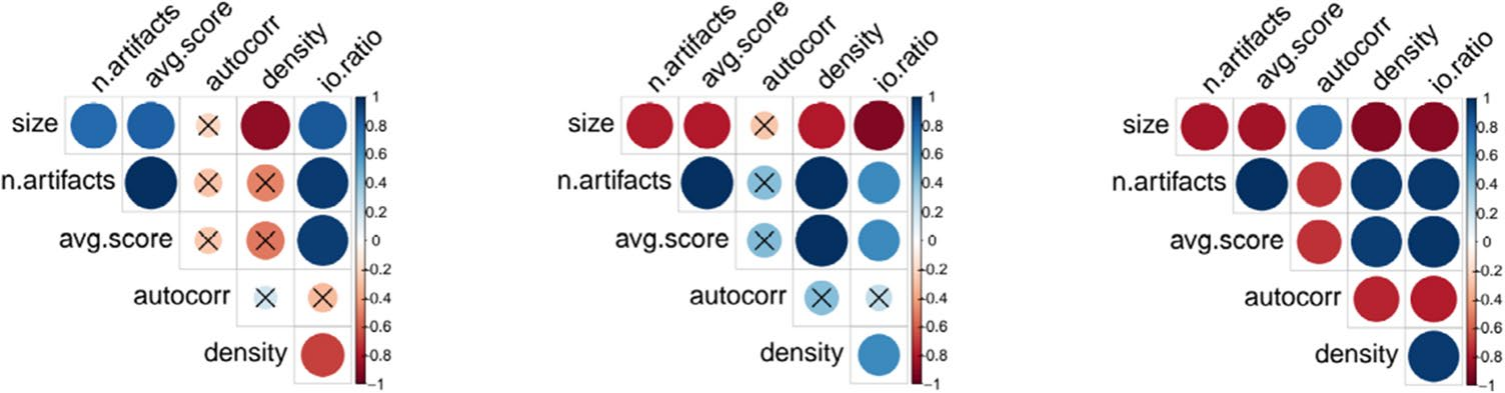
Correlation matrix for teams. One matrix for each group of teams is shown. Blue indicates positive correlation and red indicates negative correlation. Color intensity indicates the strength of correlation. Insignificant coefficients, according to *p* value $p = 0.001$, are marked with a cross. Only teams whose lifespan is ten or higher are considered.

For each team, we computed correlations between team performance metrics and team structure metrics in time. We used number of artifacts and average IC score as productivity and quality metrics. For team structure metrics, we used team size, autocorrelation, density, and ratio between inner and outer connections (see “Methods” section). For this analysis, we only considered teams whose lifespan was ten or higher. We then grouped together teams that showed similar results, forming three different groups. Groups have size of 12, 4 and 6, respectively. Figure 8 shows the correlation matrix for one representative team in each group. It can be noticed that team size is negatively correlated to performance metrics for group two and three, and positively correlated for group one. This indicates that a growth in terms of size does not always imply higher productivity or higher quality work. Autocorrelation has an influence only for the group three, correlating negativity with the performance metrics. This shows that, for these teams, high turnover is beneficial. Density correlates positively with number of artifacts and IC score for group two and three, while it has no effect for group one, showing that highly connected teams are more productive and produce higher quality work. Finally, the ratio between inner and outer connections is positively correlated to number of artifacts and IC score for all the three team groups. This means that teams that are not isolated, whose members engage in collaborations with members of other teams, are more productive and can produce higher quality work.

**Figure 9:**
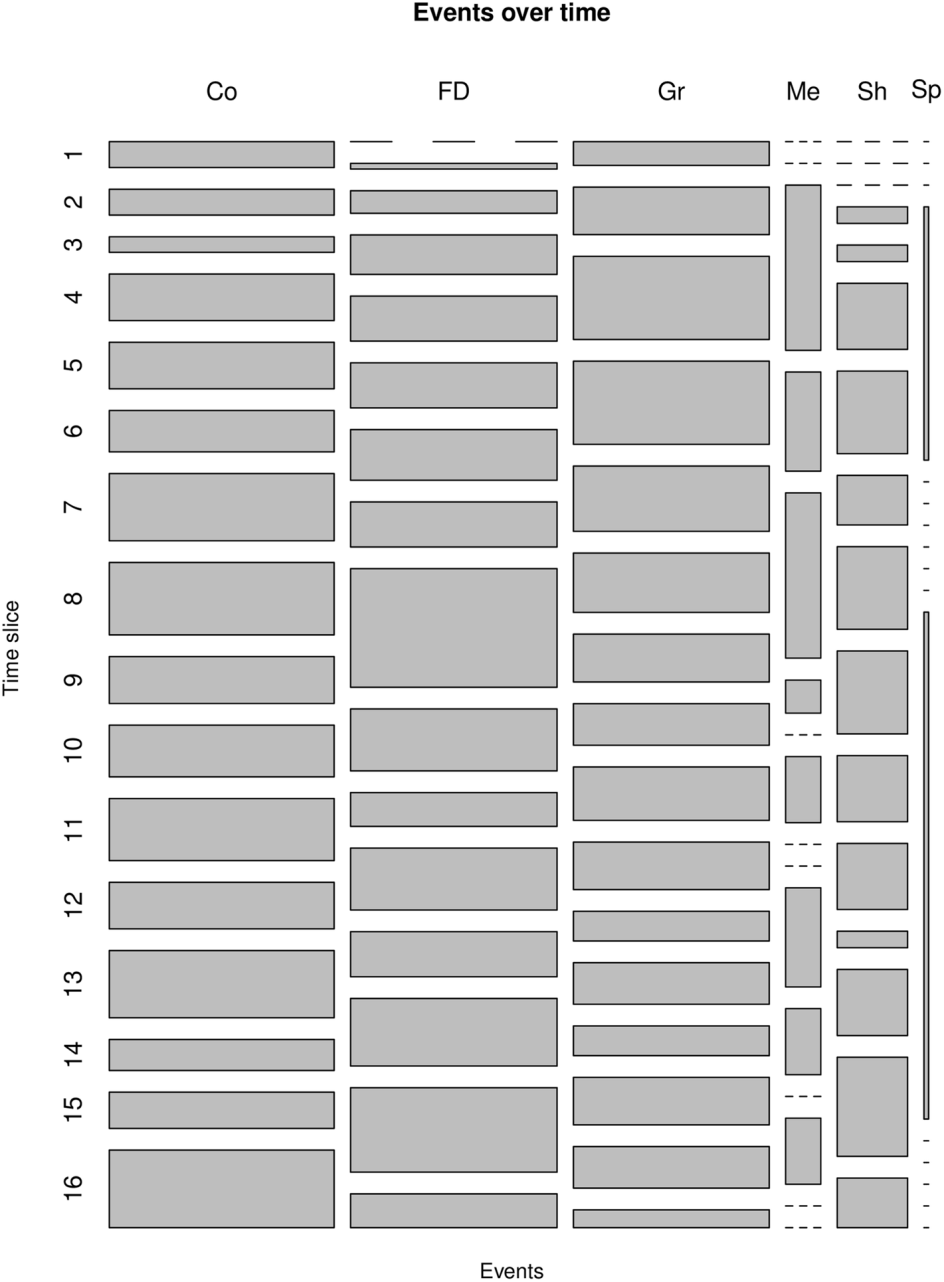
Events over time. *Co* continuing, *FD* forming or dissolving, *Gr* growing, *Me* merging, *Sh* shrinking, *Sp* splitting. For each column/event, the width of its rectangles corresponds to the frequency of this specific event over the whole time horizon. For each row/time slice, the height of a rectangle corresponds to the frequency of the event in the time slice when compered to the whole horizon.

**Figure 10:**
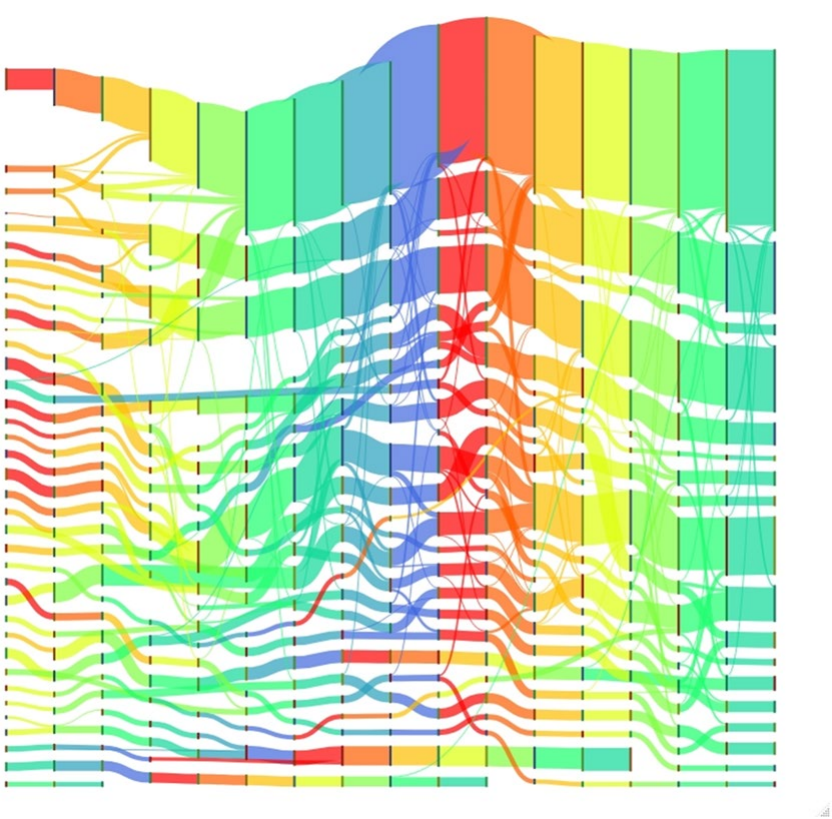
Evolution of teams over time for the NECTEC researchers network. Nodes represent communities at a certain time *t*, size represent team size. Edges represents researchers moving from one community at time *t* to another community at time $t+1$. Edge thickness represents the number of researchers. Colors represent different time transitions. Only communities of size 5 or greater are considered.

We also kept track and analysed all the changing events that affected the teams in the network such as teams growing or shrinking in size, the merging of small teams and splitting of large teams, and the birth and death of teams. Figure 9 shows all these events found in between time slices. Continuing and growing events are the most frequent in general, while merging, shrinking and splitting are more rare. The portion of forming and dissolving events corresponds to new projects starting off and terminating. Finally, Fig. 10 gives a more visual perspective on the evolution of teams. It shows all teams of size 5 or larger, and how team members move from one team to another. It can be noticed that teams are generally growing over time. Small teams merge to form bigger ones and big teams maintain or grow their size over time.

## Discussion

In this study, we used a dataset from the National Electronics and Computer Technology Center (NECTEC) in Thailand, where researchers collaborate on different projects to produce a range of artifacts (intellectual properties, prototypes and scientific articles). We build a collaboration network where researchers are connected if they worked together on one or more artifacts.

First, we analyzed the distribution for some of the attributes of the NECTEC researchers networks, showing the skewed distribution of quantities related to performance such as number of artifacts produced, IC score and career length. We focused on measuring productivity and quality of research and development, while linking these metrics to the structure of the collaboration network. We have found that researchers that cover more central positions in the network, reflected by high betweenness centrality, are more productive. More productivity indicates e.g. high number of artifacts produced, engagement in multiple projects or longer career span. At the same time, centrality metrics are not found to be correlated with average IC score, which measure quality of work rather than quantity. On the other hand, we found that researchers who have more collaborators, reflected in higher degree centrality, are more productive, but their average IC score is lower, therefore producing lower quality work. These results are in agreement with the work of Uddin, Hossain and Rasmussen who linked betweenness and degree centrality to productivity metrics^19^. These results are also in partial agreement with the findings of Feng and Alec^14^, who showed that researchers engaging in more collaborations have a longer career and higher citation count. Using orbit analysis, we showed how triangle-like graphlets are more represented than star-like graphlets in the researcher network, meaning that transitivity, more than preferencial attachement, is the main force that drives researchers to connect. In other words, researchers that are choosing their collaborators prefer a common peer to a highly skilled person. Using correlation analysis, we also found that researchers who cover more central orbits withing a certain graphlet are more productive, result that is in agreement with the latter analysis.

For what concerns teams, we analyze the distribution of team size and lifespan over time for the NECTEC researchers network, showing that the network is composed of a few large teams, while the majority of the teams are small (i.e. less than ten members). Team lifespan, instead, shows a bimodal distribution, with most teams having either a short or long lifespan. We looked at the evolution of teams over time by tracking size (team structure metrics), as well as artifact and average IC score produced by all researchers (team performance metrics). We showed that the tracking of this quantities allows to identify teams that produce high quality work, as well as to show teams’ turnover, particularly newcomers/experienced teams members that join/leave a team. We also computed correlation between team performance metrics (number of artifacts and IC score) and team structure metrics (team size, autocorrelation, density, ratio between inner and outer connections). We found that highly dense (connected) teams are more productive and produce higher quality work. This result is in agreement with Reagans and Zuckerman’s work^28^, who confirmed the closure view of social capital, stating that teams that experience more frequent communication among their members (higher density) can achieve higher productivity. High turnover is also beneficial and the result is in agreement with the work of Palla et al.^25^, who showed how large teams last longer and perform better when turnover is high. Furthermore, teams that are not isolated, whose members engage in collaborations with members of other teams, are more productive and can produce higher quality work. For what concerns team size, a larger team does not necessarily imply higher productivity or quality. Finally, we tracked all changing events affecting teams in time, showing that teams are mostly growing in size, while other events like splitting an merging of teams are more rare.

### Originality of the study and limitations

What makes this study original is the analysis based on a score that measures research quality rather than quantity (e.g. number of output and citation count), as well as a new set of structural metrics that help identify features of teams that are linked to success. Even though the analysis is limited to a rather small dataset, we believe the dataset is rich in information, especially for the availability of a quality score, which is often neglected or is just not available for analysis in other studies. Nonetheless, the methodology adopted can be extended to larger datasets/networks. The key findings of this study indicate that the success of a research institute needs to be assessed in the context of not just researcher or team level, but also on how the researchers engage in collaboration as well as on how teams evolve.

### Suitability and shortcomings of IC score

The IC score is a predefined quality indicator that is arbitrarily assigned to each artifact produced at NECTEC, hence not based on any formula. This circumstance makes it different from other related scores that are normally used to assess performance, such as number of publications and number of citations (e.g. *h*-index). Also, given the subjective nature of the IC score, this measure is likely subject to human bias. To complement the predefined IC score, we have used centrality metrics such as betweenness and closeness centrality in this study. These metrics can assess performance from an orthogonal direction compared to the IC score and the results are not simply based on the number of artifacts or collaborations.

### Team identification

Two definitions of teams have been used in this study. The first one is human-made, where a team consists of a set of people assigned to work on the same project. The second one is based on the result of a community detection algorithm on the NECTEC researchers network that detects network-based communities that can be understood as teams. These two approaches on how to capture team structures are valid and complementary methods, where one approach reveals team properties which cannot be found easily in the second approach (and vice versa).

### What managers can do to improve collaborative research?

Higher education institutions are becoming increasingly dependent on research^26^. High competition levels are beneficial for achieving high quality research, but can also bring tensions that, at institution and researcher level, could prevent researchers from engaging in collaborations. New researchers would especially benefit from collaborations with more experienced researchers. It is therefore important for managers to be aware of these dynamics, assist their staff through professional support networks^15^ and research grants^26^ that favour collaborative and interdisciplinary projects. The results obtained in this article, also supported by the current literature^15,31^, shows that collaboration is beneficial to achieve a common goal. On the other hand, collaboration is a costly process in terms of time and effort. The effort of researchers to collaborate without real commitment to achieve a common goal may result in failure. Managers must be able to understand the complexity of this process and lead research teams towards the achievement of common goals though collaboration. Is is also important to support collaborative research between different institutions. Managers should encourages these collaborations that could be beneficial for the sharing of expensive research facilities and resources^26^.

## Methods

### Dataset

The dataset includes information about projects carried on at the NECTEC institute for a 9 year period, from 2009-10-03 to 2018-07-26, for a total of 553 projects. Within each project, researchers collaborate to produce three different types of artifacts: scientific articles, prototypes and intellectual properties (IP). The dataset contains 1202 records for articles, 459 for prototypes and 631 for IP, for a total of 8531 collaborations. Time information is also included in the dataset, as filing date, for each artifact. The dataset is publicly available at https://github.com/apivadee/research-collaboration.

### Building the network

The constructed collaboration network represents researchers as nodes, that are connected to each other if they have collaborated to produce one or more artifacts. The NECTEC researchers network includes 740 nodes/researchers and 5298 edges/unique collaborations and is undirected. Nodes are assigned the following attributes:

- n.artifacts: number of artifacts produced by a researcher.
- n.projects: number of projects a researcher has participated in.
- score: total IC score assigned to all artifacts produced by a researcher, weighted by their contribution.
- percent.contrib: average percentage of contribution of a researcher for all artifacts they have worked on.
- start.career: start of a researcher’s career, i.e the filing date of the first artifact produced by a researcher.
- end.career : end of a researcher’s career, i.e. the filing date of the last artifact produced by a researcher.

Edges, instead, have the following attributes:

- n.artifacts: number of artifacts that two researchers have collaborated on. This quantity is normalized by the total number of artifacts produced by both researchers.
- n.projects: number of common projects that two researchers have participated in. This quantity is normalized by the total number of projects that the two researchers participated in.
- contribution.symmetry: indicates how much, on average in a collaboration, one researcher contributes to the production of their artifacts compared to the other. It ranges from $-\,1$ to 1 in case only one researcher contributes to all the work, and 0 in case of equal contribution.
- start.date: start date of a collaboration, i.e. the filing date of the first artifact produced by two researchers.
- end.date: end date of a collaboration, i.e. the filing date of the last artifact produced by two researchers.

A dynamic version of the network previously discussed is built. Given a sliding time window of length *d* and a time step *s*, the static network is “sliced” and a subnetwork is produced for each time slice. For example, if you consider the time interval [01/01/2008, 01/01/2018], a time window of 3 years and a time step of 1 year, the subnetworks will include all nodes that are active in the interval [01/01/2008, 01/01/2011], [01/01/2009, 01/01/2012], ..., [01/01/2015, 01/01/2018]. A node is considered active in a certain time slice if its starting time or ending time falls within the interval. The dynamic version of the NECTEC researchers network, in particular, consist of seventeen time slices.

### Performance metrics

The Intellectual Capital (IC) score, defined by NSTDA, is used to define the capital level of each R&D output within the NECTEC. It is assigned to each artifact and divided among researchers that worked on it, depending on their percentage of contribution. We have defined two different performance metrics: productivity metrics are related to the amount of work of a researcher, while quality metrics are related to the impact that a certain work has. As productivity metrics, we used number of artifacts produced by a researcher, number of projects that a researchers have joined, career length and number of collaborations. As quality metrics, we used the average IC score.

### Network metrics

Global network metrics are metrics computed over the entire network. Among these metrics there are diameter, average path length and clustering coefficient. The diameter is the longest shortest path between any two nodes in the network, while the average path length is the average shortest path between any two nodes in the network. These two metrics represent how easily information can travel through the network. Clustering coefficient is computed as the ratio of the number of triangles and the connected triples in the network.

Local network metrics are metrics computed on single nodes. Among these metrics there are local transitivity and centrality metrics such as degree, betweenness and closeness. The local clustering coefficient is the ratio of the triangles connected to a node and the triples centered on the node. This metric is related to the concept of transitivity. In the collaboration network, if researcher A is connected to researcher B and researcher B is connected to researcher C, what is the probability that researcher A is connected to researcher C? The degree centrality consists in the number of direct connections of the node and represents the number of collaborators of a researchers. The betweenness centrality of a node is computed as the number of shortest paths between any couple of nodes in the network that pass through the node. It represents how critical is the position of a researcher in the network for the transmission of information. For example, nodes with high betweenness often serve as bridges between different communities. The closeness centrality is computed as the average shortest path between the node and any other node in the network. It represents how far information has to travel from a node to reach the entire network.

### Orbits analysis

**Figure 11:**
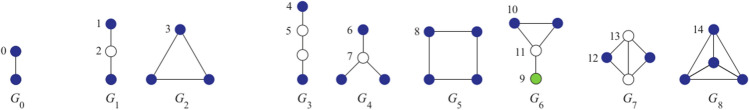
Graphlets up to 5 nodes with their relative orbits.

Graphlets are small connected graphs. Graphlets have been used in social sciences (Holland Leinhardt^32^) to study local connectivity patterns and compare networks. In the context of collaboration networks, for example, they have been used to analyse differences in collaboration patterns for male and female researchers, as well as researchers of different ethnicity, in Computer Science (Van Herck and Fiscarelli^33^). In biology, instead, they have been used to identify clusters in biological networks for protein prediction (Milenković and Pržulj^34^) and cancer gene identification (Milenković et al.^35^). The simplest graphlet has size two and consists of two nodes connected by an edge. There are then three different graphlets with up to 3 nodes, 9 with up to 4 nodes and 30 with up to 5 nodes. Nodes within a specific graphlet are not always identical. Different orbits can be identified, which represent the role of the node withing the graphet. For example, in graphlet $G_4$ (star), one represents the center and the rest of the nodes are at the periphery. All graphlets and orbits up to size four are shown in Fig. 11.

Some graphlets are characteristic of certain type of networks. For example, graphlet $G_2$ (triangle) is more likely to be found in social networks, due to high transitivity^36^. In the same way, nodes that have different “roles” in the network will have higher orbit counts for specific orbits. Example of networks that does not show high transitivity are non-social networks such as the World Wide Web^2^ and food networks^37^. It is interesting to count, for each node, the number of times it appears to be part of a specific orbit. This way, each node can be characterized by a vector of orbit counts that indicates the configurations that it is part of. Classically, a null model is used as reference and the following formula is then used: $\frac{O_i - O_i^{\mathrm{NULL}}}{O_i + O_i^{\mathrm{NULL}}}$, where $O_i$ represents the average count, over all nodes, of orbit number *i* and $O_i^{NULL}$ represents the same quantity computed with the null model. Two null models have been used. The first one is the Watts–Strogatz model^38^. It can generate small world networks that, similarly to real-world networks, show low average path length and high clustering coefficient, due to the rewiring mechanism used. The second one is the Barabási model^39^. It can generate scale-free networks whose degree follows a power-law distribution, due to the preferential attachment mechanism used to build the network.

### Team dynamics

Team dynamics or social groups dynamics is the analysis of team evolution. There has been different definition of groups^40,41^, coming from different disciplines. In Social Network Analysis, a group is defined as a set of actors that are highly connected to each other, when compared to the rest of the network^3^. This is a criterion for group existence rather than a proper definition of group. Therefore, depending on the scenario and needs, different definitions can be considered^42^. The social groups evolution problem can be decomposed into different steps:

- *Temporal network creation*: a temporal network is created by slicing the static network and extracting a network for each time slice. Networks can be generated by selecting the subset of nodes or edges active at a certain time (Palla, Barabási and Vicsek^25^) or within a time window. Time windows can be distinct (Palla, Barabási and Vicsek^25^) or overlapping (Gao et al.^27^) and their size can be constant or adapted to include a fixed number of nodes or edges.
- *Group identification*: for each network, groups are identified. Groups can be disjoint (Gao et al.^27^, Bródka, Saganowski and Kazienko^42^) or overlapping (Palla, Barabási and Vicsek^25^, Bródka, Saganowski and Kazienko^42^). Also, networks can be treated independently (Palla, Barabási and Vicsek^25^, Bródka, Saganowski and Kazienko^42^) or the group identification at time *t* can take into account the groups found at time $t-1$ (Gao et al.^27^).
- *Group tracking*: the evolution of groups is tracked by matching groups at consecutive time slices. Methods are based on similarity measures between groups (Bródka, Saganowski and Kazienko^42^), central nodes identification (Gao et al.^27^) or other methods that are suited to a specific community detection algorithm (Palla, Barabási and Vicsek^25^).
- *Event identification*: each matching between groups at consecutive time slices is associated to an event. Palla, Barabási and Vicsek^25^, as well as Bródka, Saganowski and Kazienko^42^ proposed seven different event types:
  - *continuing*: a group continues its existence when two groups at consecutive times are identical or almost;
  - *growing*: a group at time *t* grows when few nodes join the group, making its size slightly larger at time $t+1$;
  - *shrinking*: a group at time *t* shrinks when few nodes leave the group, making its size slightly smaller at time $t+1$;
  - *merging*: several groups at time *t* cease to exist and merge to form a new group at time $t+1$;
  - *splitting*: a group at time *t* ceases to exist and splits to form several new groups at time $t+1$;
  - *forming*: a new group forms when a group, that did not exist in time window *t*, appears in time window $t+1$;
  - *dissolving*: an existing group dissolves when a group, that existed in time window *t*, does not exist anymore in time window $t+1$;

In this paper, two definitions of groups are considered. The first one is related to the projects: a group is identified by all the people working on the same project. In this case groups can overlap, since one person can work on several projects at the same time. The second definition is related to the outcome of a community detection algorithm. Using a community detection algorithm whose output is a disjoint set of groups will give a significantly different outcome when compared to the first one, i.e. a single group will contain researchers working on two different projects, instead of two overlapping groups.

For this article, we used a sliding time window with length *d* and step *s* such that a temporal network with 17 time slices is generated. As group identification method, we decided to use MemLpa^43^, a community detection method based on label propagation. In this case, communities are disjoint and the outcome of the algorithm at time *t* depends on the groups found at time $t-1$. As group tracking and event identification methods, we used the ones described by Bródka, Saganowski and Kazienko^42^, that are based on a similarity measure called inclusion. According to the experimental analysis in their work, the optimal values for $\alpha$ and $\beta$ are between 0.5 and 1. Using two different values for these parameters would favour the identification of growing/merging events over shrinking/splitting events or vice versa. Therefore, the parameters we used in our experimental analysis were set to $\alpha = 0.5$ and $\beta = 0.5$, and communities with size five or lower were omitted.

### Team metrics

Two different type of metrics have been defined for teams: team performance metrics and team structure metrics. Team performance metrics include the number of artifacts produced by all team member (productivity metric) and the average IC score of all team member (quality metric). These quantities are normalized by team size. Team structure metric include team size, autocorrelation, density and ratio between inner and outer connections. Autocorrelation measures teams’ turnover, hence the rate at which researchers join and leave a team. It is computed as the intersection between a team at time *t* and time $t+1$. Low/high autocorrelation indicates steady/dynamic teams. Density indicates the density of connection within a team. It is computed as the number of connections between team members and the total number of possible connections. Low/high density indicates sparse/close-knit teams. The ratio between inner and outer connections measures in what extend team members engage in collaboration with members of other teams. It is computed as the number of edges between team members and non-team members, divided by the sum of team members’ degree. A low/high ratio indicates the tendency of teams to be closed/open. For each team, these metrics are computed for each time slice of their lifespan.
