# Author Correction: Interplay between success and patterns of human collaboration: case study of a Thai Research Institute

**DOI:** 10.1038/s41598-021-88100-2

**Published:** 2021-04-15

**Authors:** Antonio Maria Fiscarelli, Matthias R. Brust, Roland Bouffanais, Apivadee Piyatumrong, Grégoire Danoy, Pascal Bouvry

**Affiliations:** 1 Luxembourg Centre for Contemporary and Digital History (C2DH), University of Luxembourg, Esch-sur-Alzette, Luxembourg; 2 Interdisciplinary Centre for Security, Reliability and Trust (SnT), University of Luxembourg, Esch-sur-Alzette, Luxembourg; 3 Department of Mechanical Engineering, University of Ottawa, Ottawa, Canada; 4 Department of Computer Science, FSTM/DCS), University of Luxembourg, Esch-sur-Alzette, Luxembourg; 5 NSTDA Supercomputer Center (ThaiSC), National Electronics and Computer Technology Center (NECTEC), Pathum Thani, Thailand

Correction to: *Scientific Reports* 10.1038/s41598-020-79447-z, published online 11 January 2021

The Funding section in this Article was omitted. The Funding section should read:

“Supported by the Luxembourg National Research Fund (10929115).”

